# Incentives and gender in a multi-task setting: An experimental study with real-effort tasks

**DOI:** 10.1371/journal.pone.0213080

**Published:** 2019-03-14

**Authors:** Zahra Murad, Charitini Stavropoulou, Graham Cookson

**Affiliations:** 1 Economics and Finance, University of Portsmouth, Portsmouth, United Kingdom; 2 School of Health Sciences, City University of London, London, United Kingdom; 3 Office of Health Economics, Southside, London, United Kingdom; Groupe ESC Dijon Bourgogne, FRANCE

## Abstract

This paper investigates the behavioural effects of competitive, social-value and social-image incentives on men’s and women’s allocation of effort in a multi-task environment. Specifically, using two real-effort laboratory tasks, we investigate how competitive prizes, social-value generation and public awards affect effort allocation decisions between the tasks. We find that all three types of incentives significantly focus effort allocation towards the task they are applied in, but the effect varies significantly between men and women. The highest effort distortion lies with competitive incentives, which is due to the effort allocation decision of men. Women exert similar amount of effort across the three incentive conditions, with slightly lower effort levels in the social-image incentivized tasks. Our results inform how and why genders differences may persist in competitive workplaces.

## Introduction

Incentives in multi-task settings have received increasing attention in recent years. More employers than ever before require employees to multitask between different job responsibilities, a trend that has increased with the economic downturn as a means to save on labor costs [1–3]). Multi-tasking is evident in academic jobs, where university lecturers are involved in teaching, research and administrative duties [4], [5]. Similarly, most clinically active surgeons in medical centers are required to conduct research as well as examine patients and perform clinical procedures [6]. It has been argued that in these multi-task settings gender inequalities prevail: leading figures from Cambridge University in the UK have recently argued that the lack of women in top academic positions may be attributed to the way different job tasks are rewarded [7]. Academic promotions, they claim, are based on rigid and highly competitive research outcomes, such as publications and research grants, and much less on teaching and public engagement. Therefore, the criteria for success may benefit men more than women. Motivated by this observation, our study explores how various incentive schemes affect effort distortion across multiple tasks and how incentive effects are determined by the employee’s gender and economic preferences.

Previous evidence on singleton tasks has shown that there are indeed gender variations in how men and women respond to different types of incentives. Under competitive incentive schemes men have been shown to perform better than women [8], [9]. This result is particularly strong for tasks that are male-stereotyped indicating the importance of stereotype threat [10]. Competitive incentives have long been utilized in the private sector with emphasis given to relative performance evaluations. Increasingly governments have been arguing for the adoption of relative performance-related-pay schemes in public services such as healthcare, education, law enforcement and the civil service [11]. In contrast, women tend to perform better when they are given social-value incentives [12], [13]. These types of schemes are more prevalent in the public and non-profit sectors but are also becoming a feature in the private sector through firm engagement in corporate social responsibility and philanthropic projects.

Despite the growing evidence on gender differences in singleton settings, there is no empirical evidence on the effectiveness of competitive, social-value and social-image incentives on effort allocation levels for each gender in a multi-task setting. This remains one of the biggest gaps in the literature of multitasking, and an important one given the growing use of multi-task working environments [14–16]. An editorial note by [17] calls for more research on heterogeneous effectiveness of incentives with a special emphasis on demographic characteristics such as gender, ethnicity, age and nationality. Yet, these type of studies are still limited in multi-task settings.

A number of studies from single-task settings have focused on one incentive type at a time. It has been shown that gender differences under competitive payment schemes [8], [9], [18] are due to differences in risk preferences, confidence [19–21], aversion to receive relative performance feedback and willingness to compete [22], [23]. Women have been shown to be more motivated by social-value generation compared to men [12], [13], [24], [25] due to differences in pro-sociality and attitudes towards social and non-profit organizations [26–29]. There is varied evidence of gender differences in response to social-image incentives. [3], [30], [31] show that men are more motivated by social-image concerns than women which has been explained by the evolutionary motivation of men to raise their social-status [32], [33]. More recent evidence from field and lab experiments [34–37], closely related to our design of social-image incentives, found no evidence of higher performance of men compared to women in public award/ranking treatments. Our study thus closely link to this strand of literature, but investigates the effectiveness of incentives for each gender as an allocation decision in a multi-task setting: specifically we aim to investigate the effort decisions in the presence of another task, which provides a clear opportunity cost of effort in a task the additional incentive is applied to.

We use experimental methodologies to reach our aim of cleanly comparing the effectiveness of incentives by gender and economic preferences. The multi-task setting that we consider involves working on two distinct tasks under a restricted work time, which induces substitutability between the tasks. We pre-test five different real-effort tasks to choose the two with the most similar characteristics. We then use these two tasks where one of the tasks is always incentivized by a piece-rate in all treatments. In a between-subject design we vary the incentives applied to the other task. In the control condition, we use fixed pay and in three treatment conditions we use competitive, social-value and social-image incentives.

We find strong evidence that incentives have differential effects on the effort allocation decisions of men and women. All three types of incentives significantly distort effort allocation decisions towards the task to which they are applied on compared to the piece-rate paying second task. Subjects earn significantly less in the social-value and social-image treatments emphasising the social preferences of subjects and their willingness to sacrifice some of their earnings for a social cause. The highest distortion of effort across the tasks is in the competitive incentive treatment, which is solely due to the effort allocation decisions of men. Further we observe that men’s effort allocation under the social-image incentives is higher compared to the one under social-value incentives, underlining the importance of social-image concerns for men. The effort allocation decision of women, on the other hand, is the same across all three incentive treatments, with a marginally lower contribution under the social-image incentives compared to the one under social-value incentives which can be explained by the previously observed ‘wallflower effect’ [38]. We find limited evidence of economic preferences determining the effectiveness of incentives: only the attitudes towards donating to social causes significantly predicts effort allocation in a social-value generating task away from piece-rate paying task.

In contrast to the extant experimental studies in multi-task settings, we increase the external validity and generalizability of our results by designing an experiment with *real* effort rather than *chosen* effort tasks. A similar line of research was undertaken by [16], [39] who also used real effort tasks and online experiments to increase the external validity of their studies. However, both of these studies use task *complementarity* to study the interaction between the performance-related private and social incentives and the effort allocation decisions. One of the most similar designs to ours is [40] who also use two tasks, keeping incentives in one task constant and varying incentives (piece-rate, team and tournament) in the other task, to explore how incentives affect effort allocation decisions. They find that competitive tournament incentives produce more variable and higher effort levels followed by team incentives and piece-rate incentives. [15] is another study investigating the effectiveness of incentives across multiple real effort tasks. Their study finds that public visibility of relative performance feedback has a significant effect on effort allocation between the tasks: feedback improves subjects’ effort in total but distorts effort allocation towards the task in which performance feedback is publicly visible. While [40] do not report any results pertaining to gender, [15] report that they do not find any gender differences. The main reason for this could be that performance-related feedback affect both genders similarly. We discuss further our findings in the light of previous studies and implications in the concluding section of the paper.

## Experimental design

### Basic experimental design

The experiment consisted of two parts, a single-task and a multi-task part, both of which involved working on the slider task and the counting zeros task. The selection of these two tasks was based on a pre-test experiment, which was conducted among 18 subjects. The pre-test experiment explored the within-subject correlations between five real-effort tasks. Five tasks, the circle task [41], the counting zeros task [42], the ball-catching task [43], the slider task [44], [45] and the number adding task [18] were presented to subjects in a random order. Based on a within-subject analysis, we found that the highest correlation was between the slider task and the counting zeros task both in terms of actual performance and perceptions of the difficulty level. Aiming for characteristically very similar tasks to control for heterogeneous preferences for and performances in each task, we selected these two tasks as the most suitable for the purposes of our main experiment. The experiment was submitted for ethical review and was granted favorable opinion by the University of Surrey Ethics Committee (UEC/2015/042/FBEL). Written consent was obtained from all participants and the data was analyzed anonymously.

In the single-task part of our experiment, subjects sequentially received instructions about the two tasks they had to complete and had 5 minutes to complete each task. The order of the presentation of the two tasks was randomized to avoid any potential order effects. They had to complete each task separately and the performance in each task served as a control for heterogeneous abilities. At the end of a task, subjects received feedback on the number of correctly positioned sliders and completed counting zeros tables, which eliminated any potential uncertainty in subjects’ knowledge of their absolute ability levels. They were paid a piece rate of £0.10 per correct completion both in the slider and counting zeroes tasks, and the total earning in the single-task part was the sum of piece-rate earnings from both tasks.

After the single-task part, subjects completed a mid-study questionnaire that elicited demographic information (gender, age, and nationality) and self-reported economic preferences (general risk attitudes, competitiveness, confidence and attitudes towards donating to charities and social institutions) on a 7-item Likert-scale. By eliciting a number of demographic and psychological constructs we aimed to remove the salience of gender that could activate gender-related stereotypes and eliminate any possible experimenter demand effects [46]. The main reason for using mid-study rather than end-of study questionnaire was to ask subjects to choose a charity organization that they regularly donate to or whose activities they support from a list of charities offered. They could also fill in the charity of their liking that was not on the list. The chosen charity was then used in the next part of the experiment, where we induced social-value incentives as donations to chosen charities. Additionally, we chose to elicit the four economic preferences as they were identified by the previous literature to predict competitiveness, social preferences and image-seeking behaviour of subjects in single-task settings. The self-reported measures of economic preferences were similar to the validated survey instruments developed by [47] who show strong correlations of the measures with many real life and laboratory decisions. At the end of the questionnaire, we asked subjects which task they enjoyed most such that they could choose either one, both or neither. This question was aimed at controlling for subjects’ personal preferences for the tasks.

After the completion of the mid-study questionnaire, the multi-task part of the experiment started. Subjects had to multi-task for 10 minutes between the slider and the counting zeros tasks presented to them on the same screen. This part of the experiment consisted of four between-subject treatments where all subjects in a session participated in the same treatment. In all four treatments, subjects had to position a minimum of 10 sliders to receive a fixed rate of £3 and additionally they could earn a piece rate of £0.10 per completed counting zeros table. If subjects positioned less than 10 sliders their earnings from the multi-task part would be equal to £0. The fixed rate of £3 for a minimum correctly completed 10 sliders served two purposes. First, it helped to control the experimenter demand effect [46] that could affect the effort allocation decision in the slider task: subjects might have felt obliged to position a certain number of sliders since the task was on their screens. Second, our design mimicked the real world multi-task work environment where one of the tasks has a minimum required work load to be completed, otherwise the worker could get fired. For example, academics are required to provide a certain number of teaching hours [48], surgeons have to see a certain number of patients in a week and manufacturing workers have to adhere to minimum quality standards.

### Experimental treatments

Across the four treatments, we manipulated whether subjects received additional incentives for their effort in the slider task. The *Baseline* condition lacked any additional incentives other than those described above and served as a control condition. In the other three treatments, we manipulated the additional incentives applied to the fixed rate paying slider task whilst keeping the piece rate in the counting zeroes task constant. In the *Charity* treatment, the slider task was a social-value generating task where subjects could earn a donation of £0.10 for a charity of their choice per each slider they positioned correctly exceeding the minimum of 10 sliders. While completing the mid-study questionnaire, subjects were not aware that we would use the chosen charities in the later parts of the experiment. This treatment measured the effect of social-value incentives on effort allocation decisions between the tasks compared to the Baseline. The Charity treatment mimicked the environment of working in corporate social responsibility or corporate philanthropy projects, where certain job tasks and projects improve societal welfare [49], [50]. It also mimicked social service jobs such as academic multitasking where academics have to undertake multiple job tasks for the university such as teaching, publishing articles, gaining research grants and administration. There is some debate in the literature whether these roles are complementary or [51], and the nature of teaching-research nexus depends on the academic discipline (soft sciences versus hard-applied sciences) and the level of teaching (undergraduate versus postgraduate) as shown by [52–53]. In situations where the academic tasks are substitutable, the effort spent on teaching has a direct third party benefit (to students) compared to research activities that are considered to have more of a private value in terms of career progression and job mobility [4]. [5] further show that in gender differences in career progression in academia hence may persist.

We assessed the effect of social-image incentives on effort allocation decision across the tasks in the *CharityImage* treatment where we designed the treatment according to [54] definition of social-image being positional, desirable and non-tradable. Social image is achieved via favourable comparison to others in socially recognized situations, hence positional. Social image is also desirable, because good social image brings along some benefit. Finally, social image is non-tradable in the sense that it cannot be directly purchased. Instead, it must be gained individually and, therefore, it must be acquired through actions that are visible. Hence, in social value generating settings, people concerned with obtaining high social image will strive to appear more generous. In addition to donations earned for every slider exceeding the minimum of 10, subjects were told that the names of the top three highest donors in a session would be publicly announced and they would be awarded with a Thank You! certificate. The certificate was signed by the project grant holder and thanked the participants for their donation. The “Thank you!” certificate is included in Appendix A in S1 File. This design choice was inspired by a field experiment [34] and a lab experiment [35] which show significant effect of public recognition and awards on effort. We chose to use a certificate award rather than the visibility of donations as our manipulation of social-image incentives to reduce the “wallflower effect” (an aversion to deviate from average behavior) previously reported in experiments [38].

Finally, in the *Prize* treatment, we measured the effect of competitive financial incentives on effort allocation decisions between the two tasks. In this treatment, in addition to Baseline incentives subjects could earn a £5 prize if their performance in the fixed rate paying slider task was in the top three performances in their session. The amount of £5 was determined so that the prize is slightly less than the total donation made in a Charity session divided by three, thus making the treatments comparable. We ran one session of the Charity treatment before the first session of the Prize treatment to identify the average amount donated in a session and calibrate its financial prize equivalent.

By comparing Charity to Baseline, we can determine how social-value generation affects effort allocation; by comparing CharityImage to Charity, we can determine whether social-image affects effort allocation and finally by comparing Prize to CharityImage we can identify the effect of competitive private monetary rewards as opposed to competitive social-value generation effects on effort allocation. After the presentation of the multi-task instructions, subjects had to answer control questions to make sure that they have understood the incentives correctly. Subjects could start the multi-tasking part only after they have answered all control questions correctly. The full questionnaire and the experimental instructions are presented in Appendix A in S1 File. Table 1 summarises the incentives used in the multi-task part.

**Table 1:** Incentives in the multi-task part.

*Baseline*: £3 fixed rate for minimum of 10 sliders Piece rate of £0.10 for counting zeros task	*Charity*: Baseline + £0.10 donation per each additional slider in excess of the minimum
*Prize*: Baseline + £5 prize for the top three slider task performers	*CharityImage*: Charity + public certificate award for the top three donors

The incentives for the counting zeros task was kept constant at £0.10 per correctly completed table across all treatments.

It is worth noting that compared to previous literature that focuses on gender differences and incentives, we employ a continuous decision variable rather than a binary [22], [25]. This is similar to more recent studies that have looked at how subjects choose the percentage of their compensation to be based on piece-rate versus competitive incentive [55] or what subjects’ willingness to accept competitive incentive is [56]. Similarly our experiment uses continuous effort allocation decision between two differently incentivized tasks and extends the incentives to social-value and social-image incentives as well as competitively incentivized tasks.

### Procedures

At the beginning of the experiment subjects knew that only one of the (single-task or multi-task) parts would be randomly selected to be paid out. By using the random incentive system to pay subjects, we controlled for income effects and other potential interdependencies between the single-task and multi-task parts. The random incentive system is a widely used experimental procedure. For a discussion of its rationale and possible limitations see [57]. At the end of the multi-task part, subjects received feedback about their performance in both parts, the amount of donations they contributed to their chosen charity (in the Charity and ChairtyImage treatments) and whether they were in the top three of the session in their slider task performance (in the CharityImage and Prize treatments). In the CharityImage treatment, we also publicly announced the participation numbers of the top three donors, made them stand up and awarded them with a “Thank You!” certificate. All subjects were then privately paid in their cubicles.

We recruited subjects through advertising our study via weekly student-union newsletter, posters and handouts distributed around the campus. In total 210 subjects participated in the experiment with 54% female and 26% from social science departments. There were 42 subjects in the Baseline (4 sessions), 51 in the Charity (4 sessions), 65 in the CharityImage (5 sessions) and 52 in the Prize (5 sessions) treatments. The number of subjects in a session varied between 10 and 15 subjects depending on the number of fifteen registered subjects to show up to the experiment. While subjects knew that the experiment was for 15 participants when they signed up, they were not aware of how many exactly showed up as we seated them in their cubicles as soon as they arrived. This ensures that perceptions of probability of winning in Prize and CharityImage treatments were not affected by the actual number of subjects in a session. The experiments were conducted at a large university in the United Kingdom and were programmed using the software Ztree [58]. We ensured that the timing of sessions for each treatment was counterbalanced to account for time and day-of-the-week effects. Subjects received £5 for participating in one-hour session and additionally earned an average of £6.2.

## Results

We first present the descriptive statistics and conduct a parametric analysis of the determinants of single-task part performance in the slider and counting zeros tasks. We then look at the multi-task part and test whether competitive, social-value and social-image incentives distort effort allocation decisions between the two tasks compared to the Baseline condition. We test whether the effectiveness of the incentives shows gender variability and how men and women respond to each incentive and whether economic preferences of individuals predict their behaviour.

### Single-task effort

In the single-task part of the experiment, the average number (standard deviation) of completed sliders was 42.8 (12.3) with a minimum of 13 and maximum of 76 sliders. The average number (s.d.) of completed counting zeros tables were 28.2 (6.2) with a minimum of 9 and maximum of 48 counting zeros tables. In Table 2, we test whether any of the observable characteristics of subjects significantly predict the performance in the tasks. We observe a significantly lower performance in the Slider task and higher performance in the Counting Zeros task by females. The result is consistent with the previous finding [44], [45] who also find lower performance of women in the Slider task; we are however not aware of previous evidence showing a higher performance of females in the counting zeros task. We also find a lower performance of older subjects in the slider task but not in the counting zeros task. These results have some potential implications on future experimental studies that use slider and counting zeros tasks in gender and age varied subject pools, especially if either of these variables are the main point of interest. We thus use subjects’ single-task performance as an individual ability control in the parametric analysis of multi-task part, which eliminates any potential issues studying the gender interaction with the effectiveness of incentives.

**Table 2:** Single-task part performance.

Dependent Variable	Slider task performance	Counting Zeroes task performance
*Female*	-7.562 (1.65)***	2.015 (0.72)**
*Age*	-0.491 (0.09)***	-0.054 (0.04)
*British*	-0.724 (1.45)	1.391 (0.89)
*RiskTaking*	0.148 (0.64)	0.225 (0.37)
*Confidence*	-1.451 (0.66)**	0.246 (0.49)
*Competitiveness*	0.490 (0.52)	0.026 (0.39)
*DonationAttitude*	-0.424 (0.66)	-0.384 (0.21)
*FavouriteSlider*	-1.163 (1.61)	-0.997 (0.65)
*FavouriteCountingZeros*	-5.388 (1.44)***	1.849 (1.19)
*CountingZeroSingletask*	0.707 (0.10)***	
*SliderSingletask*		0.206 (.03)***
*Constant*	46.02 (5.16)***	18.00 (3.34)***
*N*	210	210
Adj R^2^	0.3442	0.2370

The reported coefficients are from an OLS regression. Clustered standard errors at session level are reported in parentheses.* 10%

** 5%

*** 1% significance levels. RiskTaking, Confidence, Competitiveness and DonationAttitude are self-reported economic preference measures from the mid-study questionnaire. FavouriteSlider and FavouriteCountingZeros are dummy variables of whether subjects reported enjoying one of the tasks more than the other.

We find a significant correlation in performance between the two tasks consistent with the pre-test results that identified the Slider and Counting Zeros tasks as the most similar tasks. Absent any strategic considerations, the positive correlation between the tasks supports that there was not an endogenous conflict between the tasks per se. This enables us to exogenously induce conflict into the multi-task part of the experiment by explicitly manipulating incentives and restricting work time. We find no significant correlation between favouring a task and performance in a task. The insignificance of the correlation between favouring a task and scoring high in a task is encouraging and potentially means that personal preferences play a negligible role in exerted effort levels under piece-rate incentives. Additionally, we check whether single-task performance measures were the same across the experimental sessions and treatment conditions. We cannot reject the hypothesis that effort in the single-task part was the same across the sessions and the treatments (all *p-values>0*.*100*). Overall, the results of the single-task part are reassuring and enable us to control for individual heterogeneity in performance and personal preferences independent of any other strategic considerations when analysing the effectiveness of incentives in the multi-task part.

### Effort allocation in the multi-task part

**Fig 1:**
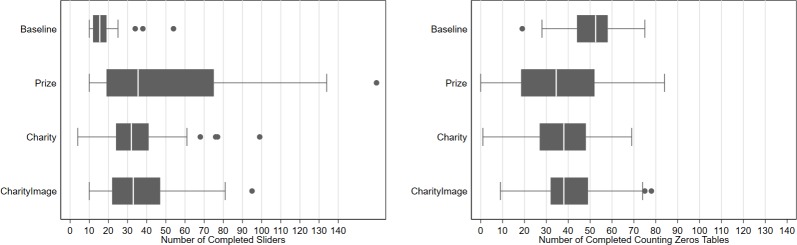
Effort allocation in the multi-task part of the experiment.

In Fig 1 and Table 3, we present the distribution of effort allocated to the fixed rate paying slider task and piece-rate paying counting zeros task in the multi-task part of the experiment across the four treatments. On inspection, the graphs show that all three incentive conditions are effective in increasing effort levels in the slider task that they are applied to. A non-parametric test on the equality of distributions shows significant differences between Baseline and the other three treatments. In the Baseline condition, the average number of completed sliders is 18 (*s*.*d*. *8*.*31*), slightly above the minimum level of required 10 sliders. Since we did not give our subjects any feedback on the number of correctly positioned sliders while they were performing the task, subjects took an extra care not to fall below the minimum threshold 10 sliders by completing on average 18 sliders. The average number of completed sliders is the highest in the Prize treatment with 51 (*s*.*d*. *40*.*23*), followed by the CharityImage treatment with 37 (21.02) and the Charity treatment with 35 (*s*.*d*. *18*.*02*) sliders. A Wilcoxon-ranksum test on the equality of distributions shows significant differences in average effort level in the slider task between the treatments and Baseline conditions (*p-value<0*.*000*). Comparing the distributions of effort levels in the slider task across the three treatment conditions, the results show significantly higher effort level in the Prize treatment compared to the Charity and CharityImage treatments *(p-value = 0*.*012* and *0*.*011*, respectively). Table A in Appendix B in S1 File presents the results of an OLS regression across the three different model specifications to test whether the differences between the treatments are robust to model specifications. We find robust effect of incentives on the effort allocation in the slider task to the inclusion of additional variables such as single-task part performance, demographics and reported economic preferences. Panel (b) of Fig 1 presents the equivalent analysis of the effort level allocated in the counting zeros task demonstrating a mirror image of the effort allocations in the slider task. The only difference in the multi-task effort levels in the two tasks is in the differences in variance between the two charity treatments. The variability in effort levels in the slider task is higher in the two charity treatments than in the Baseline whereas variability in effort levels in the counting zeros task is not significantly different between the two charity treatments and the Baseline condition. Additionally please see Figure A in Appendix B in S1 File the cumulative density function of the completed sliders in each treatment.

**Table 3:** Pairwise comparisons between the treatments.

Sliders	*Baseline*	*Prize*	*Charity*	Counting Zeros	*Baseline*	*Prize*	*Charity*
**Pairwise comparison of distributions**							
*Wilcoxon ranksum p-value*							
*Prize*	<0.001				*0*.*000*		
*Charity*	<0.001	0.232			*0*.*000*	*0*.*409*	
*CharityImage*	<0.001	0.306	0.734		*0*.*000*	*0*.*086*	*0*.*354*
**Pairwise Comparison of the means**							
*t-test p-value (2-tailed)*							
*Prize*	<0.001				*0*.*000*		
*Charity*	<0.001	0.010			*0*.*000*	*0*.*393*	
*CharityImage*	<0.001	0.019	0.504		*0*.*000*	*0*.*035*	*0*.*141*
**Pairwise comparison of variances**							
*Levene’s robust p-value*							
*Prize*	<0.001				*0*.*001*		
*Charity*	<0.001	<0.001			*0*.*354*	*0*.*007*	
*CharityImage*	<0.001	<0.001	*0*.*125*		*0*.*549*	*0*.*002*	*0*.*707*

#### Result 1

Competitive, social-value and social-image incentives are effective in increasing effort levels distorting effort levels away from piece rate paying task. The highest distortion is observed in the case of competitive incentives.

We further explore whether the variability in the effort levels in the multi-task part within each treatment is significantly predicted by subjects’ economic preferences. In Table 4, we test for heterogeneous effects of incentives on effort allocation decisions reporting the results from a linear regression; the most notable finding is that controlling for other observable characteristics, subjects with higher attitudes to donating to charities complete higher number of sliders and lower number of counting zeros tables in the Charity and CharityImage treatments.

**Table 4:** Variation in multi-task effort and self-reported economic preferences.

Dependent variable	Number of completed sliders in the multi-task part						Number of completed counting zeros tables in the multi-task part					
	Prize		Charity		CharityImage		Prize		Charity		CharityImage	
	(1)	(2)	(1)	(2)	(1)	(2)	(1)	(2)	(1)	(2)	(1)	(2)
*Risk taking*	.89	1.78	-3.08	-2.95	1.84	1.01	-1.83	-1.78	1.34	2.64	-.68	-.82
	(2.76)	(2.96)	(2.96)	(2.10)	(2.79)	(2.52)	(1.30)	(1.27)	(1.62)	(1.63)	(1.55)	(1.71)
*Confidence*	1.11	1.72	-.30	-.22	-.13	.54	-.81	-.78	-1.02	-.15	1.55	1.66
	(3.63)	(2.03)	(1.82)	(1.99)	(1.40)	(1.30)	(1.00)	(.93)	(2.48)	(1.49)	(1.45)	(1.44)
*Competitive*	15.71	17.51	-1.77	-1.75	-5.05	-8.23	-10.83	-10.74	4.56	4.72	2.22	1.66
	(16.29)	(16.73)	(7.29)	(7.46)	(7.21)	(5.10)	(8.66)	(8.76)	(5.38)	(3.97)	(4.09)	(4.63)
*Donation Attitude*	1.25	.07	2.23*	2.42*	2.27**	2.45*	-.71	-.76	-5.56**	-3.60**	-2.07**	-1.68*
	(3.52)	(3.54)	(1.27)	(1.06)	(1.08)	(1.01)	(1.75)	(1.65)	(2.06)	(1.11)	(.69)	(1.04)
*Favourite ST*	31.05*	35.06	-4.11	-3.56*	6.72	2.46	-21.75	-21.57	-.159	5.52	-5.09	-5.37
	(15.66)	(18.51)	(3.12)	(1.43)	(4.75)	(1.76)	(11.78)	(11.55)	(3.99)	(5.10)	(6.00)	(5.56)
*Favourite CZT*	5.80	11.28	4.55	5.06	-1.26	-1.14	-.38	-.12	-1.06	4.05	4.73	4.75
	(15.81)	(15.96)	(4.56)	(5.61)	(4.84)	(3.23)	(7.29)	(7.89)	(4.79)	(5.86)	(2.71)	(2.84)
*SliderSingletask*		1.26**		.08		.80**		.06		.81**		-5.37
		(.470)		(.311)		(.27)		(.20)		(.27)		(5.56)
*Constant*	11.46	-48.84	39.35*	33.90	27.69	-13.5	65.52***	62.7***	63.04*	7.20	45.37***	38.13**
	(33.99)	(31.15)	(16.49)	(35.90)	(15.88)	(21.84)	(10.97)	(15.74)	(22.16)	(30.27)	(9.88)	(13.1)
*N*	52	52	51	51	65	65	52	52	51	51	65	65
*Adj R*^*2*^	0.1881	0.3376	0.0867	0.088	0.0467	0.181	0.3135	0.3146	0.1502	0.4809	0.1378	0.1468

The reported coefficients are from an OLS regression. Clustered standard errors at session level are reported in parentheses.

* 10%

** 5%

*** 1% significance levels. ST and CZT stand for slider and counting zeros tasks respectively.

Given that we administered the questionnaire to subjects prior to them working in the multi-task part, it is highly improbable that the behaviour in the experiment affected subjects’ responses in the questionnaire. The opposite however can be true such that their responses in the questionnaire may have affected their effort allocation decisions in the multi-task part by psychologically triggering social and competitive cues. However, we find only very limited support of this. The variable measuring donation attitudes has a small effect on the effort allocation decision in the multi-task part (difference of approximately 2 sliders), while the other variables have no significant effect (e.g. contrary to expectations, reporting higher competitiveness has no effect on effort allocation into the competitive slider task).

As a measure of ability levels we find that in the Prize and CharityImage treatments, single-task performance in the slider task significantly predicts the number of sliders completed in the multi-task part. While we cannot test whether the higher number of completed sliders is due to the self-selection of more able subjects into competitively incentivized tasks or merely caused by their ability to complete more sliders in a given time, we can draw parallels with the previous literature who find similar results. There are number of previous findings of more able individuals reacting more positively to competitive environments by [34], [35] on the effect of awards in a single-task settings and van [40] on the effect of competitive prizes on effort levels in a multi-task setting. Yet a field study [59] finds that in single-task settings awards can be similarly (or even more) motivating to underperforming employees compared to overperforming ones.

#### Result 2

We find limited evidence of the effectiveness of incentives depending on preferences of subjects. Only in the Charity and CharityImage treatments, individual attitudes to donating to charities significantly predict how much effort subjects redistribute from a piece-rate paying counting zeros task to a social-value generating slider task.

### Gender differences in effort allocation

**Fig 2:**
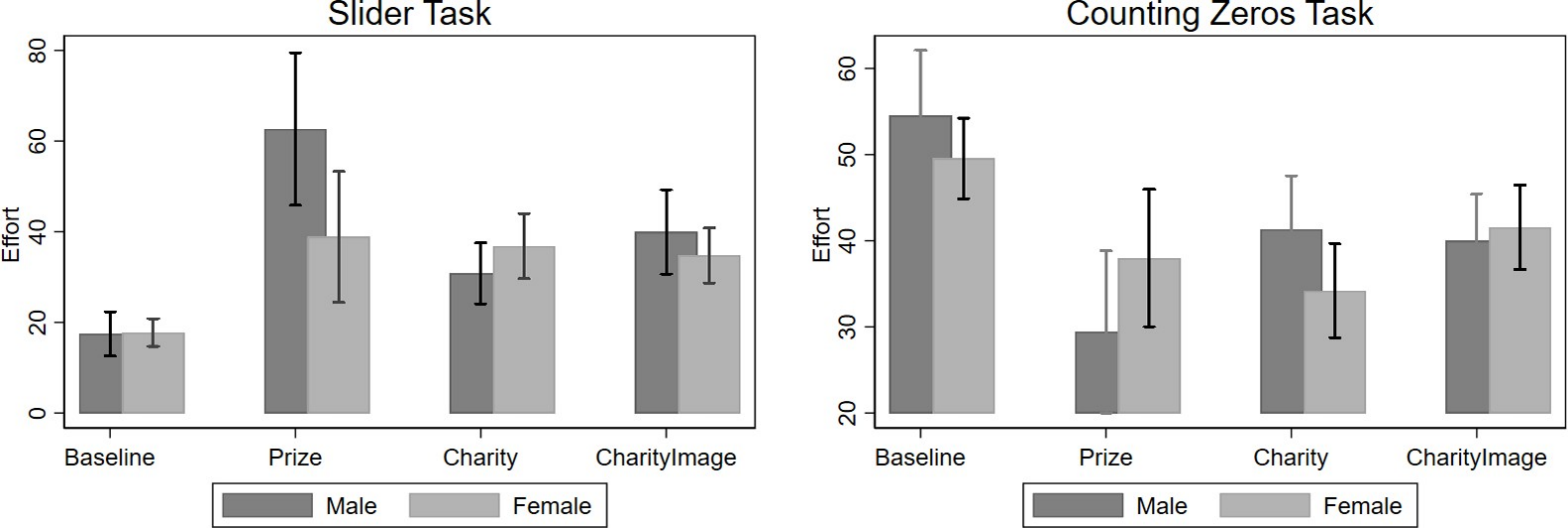
Effort allocations in the multi-task part by gender.

The visual evidence of the effectiveness of incentives on effort allocation decisions in the slider and counting zeros tasks for men and women is presented in Fig 2. In the Baseline condition, both women and men complete around 18 sliders. Men complete slightly higher number of counting zeros tables but this difference is not statistically significant: mean number of counting zeros tables completed is 54.5 for men and 49.5 for women (Wilcoxon-ranksum p-value = 0.355). The reported p-values are from a Wilcoxon-ranksum tests across the genders and treatment conditions, unless otherwise specified.

In the Prize treatment, we observe differences between men and women in the multi-task part: men and women on average complete 62.6 and 38.8 sliders (*p-value = 0*.*0315*) and 29.4 and 37.9 counting zeros tables (p-value = 0.1607), respectively. The parametric analysis of the data, reported in Table 5, provides further evidence of significant differences between men and women in their effort allocation decisions in the slider task. The coefficient of the interaction term *FemalePrize* is negative and significant under all three model specifications that control for individual single-task slider performance and other self-reported preferences of subjects. The result is consistent with the previous literature showing that when given a choice of incentives, men self-select into competitive work environments whereas women choose non-competitive payment schemes [18], [60]. Differently from these studies that focus on binary decision of men and women on whether to take up competitively incentivized task or not, our results provide evidence on how much effort to allocate between the two incentivized tasks, using a continuous dependent variable. This is similar to the recent papers [55], [56] who also use continuous measure of competitiveness and find that women have significantly lower willingness to accept competitive incentive schemes and only one-fifth of the most competitive quartile are women. Our subjects also had a choice of allocating positive amount of effort between competitively paid slider task or piece-rate paid counting zeros task and women choose to spend less effort on the slider task than men: 80% of women choose to complete less than 50 sliders while this figure is 40% for men.

**Table 5:** Incentives and gender.

Dependent Variable: Number of Completed Sliders in the Multi-task part			
	*Model 1*	*Model 2*	*Model 3*
*Female*	0.31 (2.31)	4.93 (3.08)	5.48 (3.69)
*Prize*	45.20 (8.15)***	47.11 (8.59)***	47.97 (7.91)***
*Charity*	13.34 (3.23)***	17.62 (4.61)***	17.16 (4.58)***
*CharityImage*	22.45 (3.05***	24.59(3.55)***	26.64 (4.74)***
*FemalePrize*	-24.11 (7.65)***	-25.22 (7.74)***	-25.12 (7.08)***
*FemaleCharity*	5.72 (5.89)	2.64 (6.35)	3.43 (6.44)
*FemaleChairtyImage*	-5.45 (3.43)	-6.41 (3.06)*	-8.87 (4.06)**
*SliderSingletask*		0.43 (.14)***	0.55(.18)***
*Controls*	No	No	Yes
*Constant*	17.44 (1.66)	-4.82 (7.55)	-38.56 (17.10)**
*N*	210	210	210
Adj R^2^	0.2179	0.2500	0.3004

The reported coefficients are from an OLS regression. Clustered standard errors at session level are reported in parentheses.

* 10%

** 5%

*** 1% significance levels. Controls include the economic preferences self-reported in the mid-study questionnaire and a number of subjects in a session.

#### Result 3

In the presence of a piece-rate paying counting zeros task, men exert more effort than women on the slider task when the slider task is incentivized by competitive incentives. There are no gender difference in effort levels between men and women when the slider task is incentivized by social-value and social-image incentives.

Another novel result that we observe from Fig 2 is the similarity of effort allocation decisions of women in the slider task across the three treatment conditions: 38.8 in the Prize, 36.8 in the Charity and 34.7 in the CharityImage treatments (pairwise *p-values >0*.*100*). The effort levels of men, on the other hand, in the two charity treatments is significantly lower compared to the Prize treatment: 62.6 in the Prize treatment versus 30.7 in the Charity (*p-value = 0*.*003*) and 40 in the CharityImage (*p-value = 0*.*015*) treatments. Testing for the differences in allocated effort levels in the slider task of men between the Charity and CharityImage treatments, we observe a marginally higher effort in the CharityImage treatment compared to the Charity treatment (*p-value = 0*.*074*). The parametric analysis of the number of completed sliders in the multi-tasking part (Table 6) provides further support for this result showing significant positive effect of the social-image incentives on men’s (post-estimation Wald *p-values<0*.*050*) and marginally significant negative effect on women’s (Wald *p-values = 0*.*089* in Model 2 specification) effort allocation decisions.

**Table 6:** Incentives and gender.

Dependent Variable: Number of Completed Sliders in the Multi-task part				
	*Men*		*Women*	
	*Model 1*	*Model 2*	*Model 1*	*Model 2*
*Prize*	45.20*** (8.14)	48.63*** (7.58)	21.09** (8.11)	22.20** (8.56)
*Charity*	22.45*** (3.04)	13.81** (5.38)	19.06 (2.81)	24.41*** (3.65)
*CharityImage*	13.34*** (3.23)	26.38*** (5.37)	17.00*** (1.03)	18.12*** (2.82)
*(Wald p-value)*				
*Prize = Charity*	(<0.001)	(0.001)	(0.814)	(0.846)
*Prize = CharityImage*	(<0.001)	(0.006)	(0.465)	(0.650)
*Charity = CharityImage*	(0.027)	(0.018)	(0.619)	(0.089)
*Controls*	No	Yes	No	Yes
*Constant*	17.44*** (1.65)	-33.61 (21.71)	17.75*** (0.82)	-26.71 (23.05)
*N*	92	92	118	118
Adj R^2^	0.245	0.261	0.087	0.146

The reported coefficients are from an OLS regression. Clustered standard errors at session level are reported in parentheses. * 10%

** 5%

*** 1% significance levels. Controls include the variables elicited in the mid-study questionnaire, SliderSingletask performance as a measure of ability and number of subjects in a session. The p-values for pairwise treatment comparisons are from post-estimation Wald test.

We also observe that the number of completed sliders is lower for women than for men in the CharityImage treatment (the coefficient of *FemaleCharityImage* is negative in two model specifications in Table 5). This result is in turn consistent with the previous literature showing that men are more motivated by social-image concerns and engage in more status-seeking behaviour compared to women [31]. Women on the other hand are more likely to demonstrate wallflower effect—aversion to stand out—in their cooperative and altruistic behaviour when the behaviour can be publicly observed [38].

Our results are also consistent with the findings that men are more motivated by competitive incentives where they can excel compared to their peers. The highly significant difference between CharityImage and Prize treatments for men (Wald *p-value*<0.000), though shows that a more drastic change in effort levels comes from the financial prize expectations rather than purely rank based incentive of scoring in the top three compared to the peers. We observe that while a quarter of variability in effort allocation decisions of men can be explained by the treatment conditions, only 8.7% of the variability can be explained for women which further increases to 14.6% with addition of other observable characteristics such as economics preferences. This in turn indicates that incentives can predict effort decisions of men more than of women, shedding light on how gender differences in organizations may be more rigid to be tackled with the help of incentives. We discuss this point in more detail in the concluding section.

#### Result 4

For men, competitive incentives are more effective compared to social-value, and social-image incentives to raise effort levels in the slider task. For women, competitive, social-value and social-image incentives are equally effective in raising effort levels.

An additional analysis of the heterogeneous effects of incentives separately on women’s and men’s effort allocation decisions is presented in Table 7. Most notably, we find a significant positive effect of self-reported donation attitudes on men’s effort allocation in the slider task of the Charity treatment while no such correlation is detected for women. Men’s effort donation decisions are hence, governed by their preferences for charities and social institutions. Women’s effort allocation decision, on the other hand, may be affected by perceived norms of donation rather than their own preferences for donating, a measure that we have not elicited in our experiment. Previous literature shows significant predictive power of norms on giving behaviour [61], [62], and how it differs according to gender [12].

**Table 7:** Testing for heterogeneity in effectiveness of incentives by gender.

	Prize	Charity	CharityImage
**Men:**			
*SliderSingletask*	1.43 (1.06)	0.06 (.49)	0.85 (.26)**
*Age*	0.33 (2.44)	-0.24 (.52)	0.33 (.59)
*Risk Taking*	1.38 (9.60)	0.15 (4.00)	1.77 (5.77)
*Competitive*	16.85 (35.40)	-13.87 (11.19)	-11.95 (6.15)*
*Donation Attitude*	-0.40 (7.24)	7.026 (1.82)**	1.45 (1.31)
*Favorite ST*	40.35 (40.10)	-4.74 (8.73)	6.24 (14.74)
*Favorite CZT*	1.67 (18.63)	4.95 (7.02)	-2.32 (14.83)
*NumberSubjects*	-5.79 (9.23)	2.48 (.64)*	-3.77 (2.37)
*Const*	10.53 (91.09)	-32.27 (45.49)	20.21 (40.62)
*N*	26	19	29
*Adj R*^*2*^	0.029	0.042	0.001
**Women:**			
*SliderSingletask*	1.18 (.76)	0.29 (.27)	0.87 (.59)
*Age*	1.99 (2.06)	0.49 (.95)	0.10 (.22)
*Risk Taking*	3.16 (5.02)	-5.21 (2.60)	0.61 (2.31)
*Competitive*	19.29 (11.70)	0.61 (12.05)	-5.21 (6.47)
*Donation Attitude*	2.64 (2.13)	0.90 (6.31)	4.23 (2.31)
*Favorite ST*	34.27 (17.97)	-2.92 (7.07)	3.46 (12.11)
*Favorite CZT*	20.44 (16.96)	2.51 (12.24)	-3.14 (4.87)
*NumberSubjects*	-12.49 (6.72)	-1.24 (1.69)	-0.64 (2.69)
*Const*	29.65 (82.57)	50.13 (57.44)	-16.62 (19.49)
*N*	26	32	36
*Adj R*^*2*^	0.0165	0.002	0.117

The reported coefficients are from an OLS regression. Clustered standard errors at session level are reported in parentheses. NumberSubjects is the number of subjects in a session as a measure of probability of winning the prize/award.

* *10%*

** *5%*, *** 1% significance levels.

#### Result 5

The difference in the effectiveness of incentives between men and women cannot be solely explained by their self-reported economic preferences.

## Conclusion

The incentive effects on women and men’s selection to work on one task or another is remarkably different from each other. We find that while men are more responsive to competitive and social-image incentives being applied to a fixed rate paying task, women exert similar amount of effort when competitive, social-value and social-image incentive is applied on the fixed rate paying task.

Our findings on differential effects of incentives on women’s and men’s effort levels contribute to the field studies analyzing selection effects into the public and private sector education providers [63], selection effect into more social or financial online work environments [39] and studies on incentive effects in various laboratory experiments [8], [31], [60]. While some studies find that women are more willing to exert effort in social-value generating tasks and tend to self-select into social environments more often [12], [13], [24] when they are given a choice of social-value and piece-rate generating tasks, they do not exert more effort in social-value tasks than men or than when they are given a choice between competitive and piece-rate generating tasks.

Furthermore, although publicly awarding top three donors in a lab experiment might seem artificial, the incentives in our CharityImage treatment affect exerted effort levels of men similarly positively as public recognition of employees and students analyzed in natural field settings [34]. Our results, moreover, resemble [35] findings from laboratory experiments. A marginally negative effect of public recognition on women’s performance, on the other hand, is consistent with recent findings in the laboratory experiment that investigate ‘wallflower’ effects: [38] find that women are more likely to avoid standing out when their behavior, in this case their donations to a charity, can be publicly observed. We cannot rule out that the specificities of our experimental design (i.e., awarding top three performers and public recognition in a laboratory setting) might have influenced this finding. A similar setup in an environment in which the employees’ self-esteem is more closely linked to their work might increase the positive incentive effects of social-image for men and eliminate negative incentives effects of social-image for women. However, the effects could also be reversed, for instance if the share of shy or introverted employees in the work force is sufficiently large. This implication needs further investigation.

While some studies have previously found men responding more strongly to relative performance feedback and competitive incentives [64], others find that men and women do not react differently to competitive situations per se [34–36]. Taken together these results may indicate that gender differences in competitive behavior may depend on the characteristics of the competition, such as for example, size of the prize (Gill & Prowse 2014) or the presence of a second task as in our experiment.

Similar to previous studies [34], [35], [60] we find that more able individuals react more positively to competitive environments in both of our Prize and CharityImage treatments. However, differently from previous studies [47], overall, we do not find predictive power of self-reported economic attitudes (competitiveness, risk, confidence and social) on effort levels allocated to competitive, social-value or social-image tasks. We only find that men’s attitudes towards charities and other non-profit organizations predict allocated effort levels to social-value task in the Charity treatment. This in turn, suggests the low power of self-reported measures to predict behavior in lab experiments and potentially field surveys contrary to the findings of the previous literature. This result is in itself an important contribution to the literature using self-reported non-incentivized measures of economic attitudes.

One limitation of our study is that using real effort tasks in experiments causes the experimenter to lose control of costs and benefits of the task. The output of the task depends both on the effort provided and individual abilities and skills (and in most cases in a non-linear way). This adds noise to our results and possible mitigates the effect sizes. The literature is currently split into advantages and disadvantages of using real effort tasks weighing the benefits of adding realism and external validity while preserving control and internal validity in experiments (for a short discussion see [43], [65]. Secondly, the generalizability of the results to more than two task setting is not clear. While we adopted two tasks as simple case for the current research aims, we acknowledge that with more than two tasks and thus more than two incentives, the effort allocation problem may be different. However, given our analytical framework there is no reason for a rational agent to behave differently when making a choice of effort across more than two tasks as compared to just two tasks. Not incentivizing the elicitation of socio-economic preferences is another limitation of the study which we have discussed before. Future research should investigate if the results would differ if we have more than two multi-task environment and whether economic preferences are incentivized.

## Supporting information

S1 File Supporting info. (DOCX)
