# Supplementary material for: Incentives and gender in a multi-task setting: An experimental study with real-effort tasks
Source: PLoS One. 2019 Mar 14;14(3):e0213080. doi: 10.1371/journal.pone.0213080 (PMC6417700; doi:10.1371/journal.pone.0213080)
Supplement: S1 File — (DOCX) [file pone.0213080.s001.docx]

**S1 File**

**Appendix A:**

## **Experimental Instructions**

Welcome!

You are about to participate in an experiment on decision making. You will be paid a showup fee of £2 for showing up to the experiment. You will also get £3 for completing the experiment. Additionally, you can earn money during the experiment.

This experiment will consist of several parts. The instructions for each part will be shown on your screen before the start of each part. Please read the instructions on your screen carefully and take notes if necessary as you will not be able to go back to read them.

At the end of the experiment, one of the parts will be randomly selected by the computer. Your final earnings will be determined according to your performance in the selected part. Each part has an equal chance of being selected for the payment so please pay an equal attention to each part.

Throughout the experiment you must not communicate with other participants. The use of any electronic devices is strictly prohibited. If you break these rules, you will be excluded from the experiment without receiving any fee.

You may leave at any point during the experiment if you do not wish to complete the experiment. If you leave the experiment before it is completed, you will only be paid the show up fee of £2.

Your decisions are anonymous and under no circumstances will be linked to your identity.

If you agree with these rules, you can sign and date the CONSENT FORM on your desks that you are willing to participate in this experiment and consenting to the use of your data.

If you have a question please raise your hand and someone will come to your desk to answer it.

[On screen instructions:]

**Part 1**

In this part of the experiment, you will complete two tasks: Slider task and Counting Zeros task. You will have 300 seconds (5 minutes) to complete each task. You will complete one task after the other. **If Part 1 is selected for the payment, you will be paid the sum of your earnings from both of the tasks.** You will see the detailed instructions for the Slider and Counting Zeros tasks on your screens.

If you have a question please raise your hand and someone will come to your desk to answer it. [Next Button]

**Part 1A: Slider Task**

In this task, you will be asked to position a series of sliders. The figure below shows the work screen you will use for this task. Each slider is initially positioned at 0 and can be moved as far as 100. Each slider has a number below showing its current position. You can use **the mouse or the arrow keys** on the keyboard to move each slider. You can readjust the position of each slider as many times as you wish. After you have positioned a slider, you can click the NEXT button and a new slider will be generated. Your performance in this task will be the number of sliders positioned at exactly 50 at the end of the 300 seconds. You will earn £0.10 for each slider you positioned at exactly 50. At the end of Part 1, you will learn how many sliders you have positioned correctly.

If you have a question please raise your hand and someone will come to your desk to answer it.

If you are ready, click Start the Task button.

**Part 1B: Counting Zeros Task**

In this task, you will be asked to count zeros in a series of tables. The figure below shows the work screen you will work on for this task. You will enter the number of zeros into the box below the table. After you have entered the number, you can click the NEXT button. No matter if the answer is correct or not, a new table will be generated. Your performance in this task will be the number of correctly solved tables at the end of the 300 seconds. You will earn £0.10 for each table you solved correctly. If you enter a wrong number for a table, you will earn nothing for that table. At the end of Part 1, you will learn how many tables you have solved correctly.

If you have a question please raise your hand and someone will come to your desk to answer it.

If you are ready, click Start the Task button.


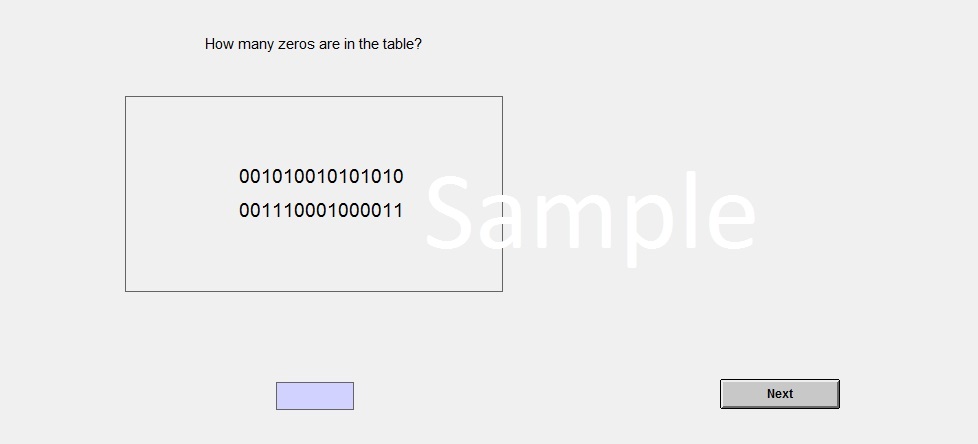


You have finished Part 1 of the experiment. You have correctly completed <#> sliders and <#> counting zeros tables. Before proceeding to Part 2 of the experiment, please answer the following questions. Your answers are anonymous and will not be linked to your identity. After you have submitted your answers you can proceed to Part 2.

**Questionnaire**

Which task did you enjoy the most? □ Neither □ Both; □ Slider Task; □ Counting Zeros Task

What is your gender? □ Male □ Female

What is your age? __________

What is your nationality?

□ British □ Other

If you are a student, what is your subject area? ______

On a scale of 1 to 7, how willing are you to take risks in general?

1 □ 2 □ 3 □ 4 □ 5 □ 6 □ 7 □

Not at all willing Very willing

On a scale of 1 to 7, how confident are you as a person?

1 □ 2 □ 3 □ 4 □ 5 □ 6 □ 7 □

Not at all confident Extremely confident

On a scale of 1 to 7, how competitive are you as a person?

1 □ 2 □ 3 □ 4 □ 5 □ 6 □ 7 □

Not at all competitive Extremely Competitive

On a scale of 1 to 7, how important it is to donate to charities and other social institutions?

1 □ 2 □ 3 □ 4 □ 5 □ 6 □ 7 □

Not at all important Extremely Important

From the list below, please select your preferred charity (the charity that you regularly donate or would like to regularly donate money to or whose activities you admire and support).

1 British Red Cross □ 2 Cancer Research □

3 World Wildlife Fund □ 4 Help the Heroes □

5 Save the Children □ 6 Student Union □

7 Other _____________ (please specify)

**Part 2: [BASELINE CONDITION]**

This is Part 2 of the experiment. In this part of the experiment, you are asked to complete both the Counting Zeros and Slider tasks on the same screen. You will have 600 seconds in total to work on both tasks. **If Part 2 is selected for the payment, your payoff will be determined as following.** You will receive a fixed fee of £3 for correctly positioning a minimum of 10 sliders. In addition to this fixed fee, you have a chance to earn £0.10 per each correctly completed Counting Zeros table. However, if you position less than 10 sliders correctly, regardless of your performance in the Counting Zeros table, your payoff in Part 2 will be £0!

To proceed to Part 2, we first need to check your understanding of the payoff structure by asking you to answer the following questions correctly. \par \par

If you have a question please raise your hand and someone will come to your desk to answer it.

1. If you complete 9 sliders and 25 counting zeros tables correctly in Part 2, and Part 2 is selected for the payment, how much will your earnings be (excluding the showup fee)? ___ (£0)

If you complete 10 sliders and 25 counting zeros tables correctly in Part 2, and Part 2 is selected for the payment, how much will your earnings be (excluding the showup fee)? ___ (£5.5)

If you complete 15 sliders and 25 counting zeros tables correctly in Part 2, and Part 2 is selected for the payment, how much will your earnings be (excluding the showup fee)? ___ (£5.5)

**Part 2: [PRIZE TREATMENT]**

This is Part 2 of the experiment. In this part of the experiment, you are asked to complete both of the Counting Zeros and Slider tasks on the same screen. You will have 600 seconds in total to work on both tasks. **If Part 2 is selected for the payment, your payoff will be determined as following.** You will receive a fixed fee of £3 for correctly positioning a minimum of 10 sliders. In addition to this fixed fee, you have a chance to earn £0.10 per each correctly completed Counting Zeros table. However, if you correctly position less than 10 sliders, regardless of your performance in the Counting Zeros table, your payoff in Part 2 will be £0!

In this part of the experiment, you also have a chance to win a **Prize of £5** in addition to your final earnings. The computer will compare your performance to the other participants’ performances in the Slider task in Part 2. If you are among the top three performers that correctly positioned the highest number of sliders in Part 2, you will earn a prize of £5 that will be added to your final earnings. You will win a Prize of £5 even if Part 2 is not selected for the payment.

To proceed to Part 2, we first need to check your understanding of the payoff structure in Part 2 by asking you to answer the following questions correctly.

If you have a question please raise your hand and someone will come to your desk to answer it.

If you complete 9 sliders and 25 counting zeros tables correctly in Part 2, and Part 2 is selected by the random draw for the payment, how much will your earnings be? ___ (£5.5)

If you complete 10 sliders and 25 counting zeros tables correctly in Part 2, and Part 2 is selected by the random draw for the payment, how much will your earnings be? ___ (£5.5)

If you complete 11 sliders and 25 counting zeros tables correctly in Part 2, and Part 2 is selected by the random draw for the payment, how much will your earnings be? ___ (£5.5)

If you complete 20 sliders and 25 counting zeros tables correctly in Part 2, Part 2 is selected for the payment and you are one of the top three highest performers in the Slider task in Part 2, how much will your earnings be? ____ (£10.5)

If you complete 25 sliders and 25 counting zeros tables in both Part 1, Part 1 is selected for the payment and you are one of the top three highest performers in the Slider task in Part 2, how much will your earnings be? ___ (£15)

**Part 2: [CHARITYIMAGE TREATMENT]**

This is Part 2 of the experiment. In this part of the experiment, you are asked to complete both of the Counting Zeros and Slider tasks on the same screen. You will have 600 seconds in total to work on both tasks. **If Part 2 is selected for the payment, your payoff will be determined as following.** You will receive a fixed fee of £3 for correctly positioning a minimum of 10 sliders. In addition to this fixed fee, you have a chance to earn £0.10 per each correctly completed Counting Zeros table. However, if you correctly position less than 10 sliders, regardless of your performance in the Counting Zeros table, your payoff in Part 2 will be £0!

In this part of the experiment, you also have a chance to earn a donation to the charity <Charity> in addition to your final earnings. We will donate £0.1 per each slider you position correctly in addition to the minimum of 10 sliders. You will earn a donation of 0.10 for each additional slider you position correctly after the 10th slider even if Part 2 is not selected for the payment. We will compare your donation amount to the other participants' donation amounts and if you are among the top three donors, your participant number will be publicly announced and you will be presented a **Thank You! Certificate** as one of the highest three donors in the experiment. We will send you an email with the receipts of donations made at the end of the study.

To proceed to Part 2, we first need to check your understanding of the payoff structure by asking you to answer the following questions correctly

If you have a question please raise your hand and someone will come to your desk to answer it.

1 If you complete 9 sliders and 25 counting zeros tables correctly in Part 2, and Part 2 is selected by the random draw for the payment, how much will your final earnings and donation be? ___ (£0 final earnings) and ____ (£0 donation)

2 If you complete 10 sliders and 25 counting zeros tables correctly in Part 2, and Part 2 is selected by the random draw for the payment, how much will your final earnings and donation be? ___ (£5.5 final earnings) and ___ (£0 donation)

3 If you complete 15 sliders and 25 counting zeros tables correctly in Part 2, and Part 2 is selected by the random draw for the payment, how much will your final earnings and donation be? ___ (£5.5 final earnings) and ___ (£0.50 donation)

4 If you complete 25 sliders and 25 counting zeros tables in Part 1, Part 1 is selected for the payment and you position 20 sliders correctly in Part 2, how much will your final earnings and donation be? ___ (£5 final earnings) and ___ (£1 donation)

5 If you complete 25 sliders and 25 counting zeros tables correctly in Part 2, Part 2 is selected for the payment and you are one of the three highest performers in the Slider task in Part 2, how much will your earnings and donation be? ____ (£5.5 final earnings), ___ (£1 donation) with a Thank You Certificate/ Without a Thank You Certificate.

**Part 2: [CHARITY TREATMENT]**

This is Part 2 of the experiment. In this part of the experiment, you are asked to complete both of the Counting Zeros and Slider tasks on the same screen. You will have 600 seconds in total to work on both tasks. **If Part 2 is selected for the payment, your payoff will be determined as following.** You will receive a fixed fee of £3 for correctly positioning a minimum of 10 sliders. In addition to this fixed fee, you have a chance to earn £0.10 per each correctly completed Counting Zeros table. However, if you correctly position less than 10 sliders, regardless of your performance in the Counting Zeros table, your payoff in Part 2 will be £0!

In this part of the experiment, you also have a chance to earn a donation to the charity <Charity> in addition to your final earnings. We will donate £0.1 per each slider you position correctly in addition to the minimum of 10 sliders. You will earn a donation of 0.10 for each additional slider you position correctly after the 10th slider even if Part 2 is not selected for the payment. We will send you an email with the receipts of donations made at the end of the study.

To proceed to Part 2, we first need to check your understanding of the payoff structure by asking you to answer the following questions correctly

If you have a question please raise your hand and someone will come to your desk to answer it.

1 If you complete 9 sliders and 25 counting zeros tables correctly in Part 2, and Part 2 is selected by the random draw for the payment, how much will your final earnings and donation be? ___ (£0 final earnings) and ____ (£0 donation)

2 If you complete 10 sliders and 25 counting zeros tables correctly in Part 2, and Part 2 is selected by the random draw for the payment, how much will your final earnings and donation be? ___ (£5.5 final earnings) and ___ (£0 donation)

3 If you complete 15 sliders and 25 counting zeros tables correctly in Part 2, and Part 2 is selected by the random draw for the payment, how much will your final earnings and donation be? ___ (£5.5 final earnings) and ___ (£0.50 donation)

4 If you complete 25 sliders and 25 counting zeros tables in Part 1, Part 1 is selected for the payment and you position 20 sliders correctly in Part 2, how much will your final earnings and donation be? ___ (£5 final earnings) and ___ (£1 donation)

5 If you complete 25 sliders and 25 counting zeros tables correctly in Part 2, Part 2 is selected for the payment and you are one of the three highest performers in the Slider task in Part 2, how much will your earnings and donation be? ____ (£5.5 final earnings), ___ (£1 donation)

**[Feedback and Payment Stage]:**

In Part 1, you completed <#> Sliders and <#> Counting Zeros tables. In Part 2, you completed <#> Sliders and <#> Counting Zeros tables.

Prize Treatment {Your performance in the Slider task of the Part 2 was <in the top three of performances. So you won a Prize of £5/ not in the top three of performances.>

Charity Treatment {We will donate £<#> to the charity <text>. Your donation was <in the top three of donations. [CharityImage Treatment {So you will be presented with a Thank You! Certificate}]/not in the top three of donations.>}

Part <#> was chosen for the payment. Your earnings for Part <#> are £#. You will be paid £<total> for participating in this experiment.

Thank you for your participation! Please wait at your desk until the experimenter approaches you with the payment.

## **Thank You! certificate in the CharityImage treatment**


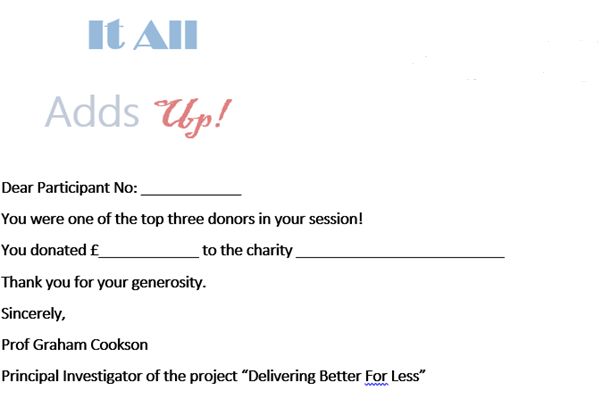


# **Appendix B: Additional Figures and Tables**

| **Table A: Predicting the Number of Completed Sliders in the Multitask Part** | | | |
| --- | --- | --- | --- |
|  | *Model 1* | *Model 2* | *Model 3* |
| *Prize* | 33.13 (8.45)*** | 34.26 (8.63)*** | 34.92 (8.64)*** |
| *Charity* | 16.94 (.90)*** | 19.85 (1.60)*** | 20.33 (3.13)*** |
| *CharityImage* | 19.42 (1.56)*** | 21.12 (2.05)*** | 22.03 (3.26)*** |
| *SliderSingletask* |  | .47 (.148)*** | .61 (.187)*** |
|  |  |  |  |
| *Prize=Charity (p-value)* | (0.0722) | (0.0940) | (0.0848) |
| *Prize=CharityImage (p-value)* | (0.1272) | (0.1360) | (0.1371) |
| *Charity=CharityImage (p-value)* | (0.1517) | (0.4208) | (0.4392) |
| *Controls* | No | No | Yes |
| *Constant* | 17.61 (.52)*** | -4.24 (6.71) | -40.06 (18.72)** |
| *N* | 210 | 210 | 210 |
| *Adj R^2^* | 0.1528 | 0.1944 | 0.2202 |
| *The reported coefficients are from an OLS regression. Clustered standard errors at session level are reported in parentheses. * 10%, ** 5%, *** 1% significance levels. Controls include the variables elicited in the mid-study questionnaire as reported in Table 2.The p-values for pairwise treatment comparisons are from post-estimation Wald test.* | | | |

**Figure A: Cumulative density function of the completed sliders in the multitask part**
